# Incidence and assessment of demography-related risk factors associated with pulmonary tuberculosis in Saudi Arabia: A retrospective analysis

**DOI:** 10.12669/pjms.38.4.5087

**Published:** 2022

**Authors:** Omar S. El-Masry, Muzaheed

**Affiliations:** 1 Dr. Omar S. El-Masry, Ph.D, Department of Clinical Laboratory Science, College of Applied Medical Sciences, Imam Abdulrahman Bin Faisal University, Dammam 31441, Kingdom of Saudi Arabia; 2 Dr. Muzaheed, Ph.D., Department of Clinical Laboratory Science, College of Applied Medical Sciences, Imam Abdulrahman Bin Faisal University, Dammam 31441, Kingdom of Saudi Arabia

**Keywords:** Pulmonary Tuberculosis, Incidence, Risk Factors, Demography, Saudi Arabia

## Abstract

**Background & Objectives:**

Tuberculosis (TB) is a public health challenge and is endemic in many countries including Saudi Arabia. The disease is a major health concern in the Kingdom because of its dynamic population as resident expatriates are mainly from high TB burdened countries and the mass influx of pilgrims every year in peak seasons for Umrah and Hajj. The objective of the current study was to evaluate pulmonary TB incidence rates and conclude the potential high-risk patients to highlight the burdened regions in Saudi Arabia for the health authorities, which could help to establish policies of infection control as necessary.

**Methods:**

We retrospectively investigated the incidence of pulmonary TB data reported by the ministry of health (MOH) in 2018. We analyzed pulmonary TB incidence data by nationality, age, gender, and region using Chi-square test to identify demography-related risk factors associated with pulmonary TB and its significance.

**Results:**

The results indicated that the incidence of pulmonary TB was significantly higher in males than in females in both Saudi and non-Saudi nationals; the number of cases was particularly high in major cities. Also, infections were mainly associated with certain age groups that were different between the Saudi and non-Saudi nationals.

**Conclusion:**

TB control seems to be facing some challenges in several regions of the Kingdom, particularly major cities. National TB Control Program (NTP) needs to continually evaluate official data to spot high risk groups and factors associated with increased incidence. This will help to improve TB control strategies to contain the disease and approaches its eradication.

## INTRODUCTION

Tuberculosis (TB) is caused by the airborne infectious pathogen, *Mycobacterium tuberculosis*.^1^ It primarily infects the lungs causing pulmonary TB and can invade other organs causing extrapulmonary TB (EPTB).^2^ TB is prevalent in all countries and distributed in various age groups.^3^ Of note,10% of healthy individuals who are infected with *M. tuberculosis* develop TB within two years.^4^ In addition, the incidence is higher among people with human immunodeficiency virus (HIV) infections.^5^

Despite of the major measures to prevent and control the spread of tuberculosis, the disease continues to be a serious global public health concern, particularly in the developing countries.^10^ In this respect, the top six TB burdened countries are India, Indonesia, China, Nigeria, Pakistan, and South Africa where infections in these countries account for 60% of new cases worldwide. India and China account for half of all global TB cases.^11^ It was also reported that pulmonary TB is the most prevalent form of the disease in the northern part of Iran.^12^ Another Middle East country with a high incidence rate as reported in 2014 is Turkey, with 22% of all reports out of 58252 recorded cases in the Middle East.^13^ Also, the global TB report that was published in 2016 ranked the gulf countries based on the incidence rate of TB, where Kuwait came first with an incidence of 200 cases/million, followed by Saudi Arabia (89/million), and then United Arab Emirates with the least incidence rate (6.8/million).^14^ Coming to the developed countries, the rates in the United States has declined remarkably to three cases/100000, which could be attributed to the advanced health care system in the country in comparison to the developing countries.^15^

Regarding mortality, it increases with the presence of comorbidities.^16^ For example, HIV and TB co-infection is an important cause of mortality in HIV patients.^10^ In addition, diabetes mellitus (DM) is associated with an increased mortality rates following TB infections.^17^ In this regard, DM is an established comorbidity that accelerates TB complications and derail its treatment. Likewise, smoking increases the risk of TB as well as its related mortality rates. To sum up, the mortality predictors include older age, low body weight, rural living, discontinued treatment, EPTB, immunocompromisation, comorbidities, and coinfections, especially HIV infections.^9^

The objective of the current retrospective study was to analyze the reported cases of pulmonary TB to comprehend the epidemiology and ascertain its association with the risk factors that may aid the National Tuberculosis Program (NTP) of the Saudi Arabia to spot high risk groups and figure out the success of infection control measures to revise their policies and measures to combat the disease.

## METHODS

This is a retrospective study that was conducted to analyze official pulmonary TB figures in Saudi Arabia cities that were reported by the MOH for the year 2018 (https://data.gov.sa/Data/en/organization/ministry_of_health). The official data figures of pulmonary TB cases were obtained and comprised the figures of 13 provinces and seven districts for Saudi and non-Saudi nationals as well as incidence amongst males and females. Cases were categorized into seven age groups, which were as follows: < 15, 15–25, 25–35, 35–45, 45–55, 55-65, and ≥ 65 years old. Also, cases were stratified according to gender, nationality, and region to compare the incidence rates between different strata of the study sample. Of note, the status of non-Saudi cases in the MOH reports was not indicated as being visitors or residents.

### Statistical Analysis:

The data was tabulated in Microsoft Office Excel and the descriptive statistics such as mean; sum and percent distribution were calculated. The comparison between the incidence rates in different age groups and gender for Saudi and non-Saudi nationals were computed by Chi Square test using IBM SPSS (Version 23; IBM Corp., Armonk, N.Y., USA). *P-value* of ≤ 0.05 was considered statistically significant.

### Ethical Approval:

This is a secondary data analysis-based study, which does not require to be ethically reviewed and approved as all data are publicly available through the Ministry of Health portal without any reference to personal identifying information of patients, for this reason, IRB approval is not applicable.

## RESULTS

### The rate of incidence:

The number of cases of pulmonary TB reported by the MOH in Saudi Arabia cities in 2018 is presented in Table-I. The results indicated that the highest number of cases amongst Saudi nationals was recorded in the capital city of Riyadh with most subjects being males (79.2%), which was also the case for Jeddah where males represented most cases (81.2%). Overall, the occurrence of infections in Saudi males was higher than that in females, except in Jazan, wherein the percentage of infections in females was 58.2%.

**Table-I:** Region-wise incidence of Pulmonary Tuberculosis among Saudi and non-Saudi population (2018).

Region	Saudi			Non-Saudi			Total Cases
	
	Male	Female	Total	Male	Female	Total	
Riyadh	255	67	322	241	97	338	660
Makkah	60	27	87	66	41	107	194
Jeddah	243	56	299	332	119	451	750
Taif	14	06	20	16	05	21	41
Madinah	42	19	61	43	17	60	121
Qaseem	13	06	19	15	05	20	39
Eastern province	56	20	76	146	28	174	250
Al-Ahsa	06	02	08	16	05	21	29
Hafr Al-Baten	10	02	12	10	04	14	26
Aseer	15	14	29	34	03	37	66
Bishah	08	05	13	05	05	10	23
Tabouk	03	03	06	15	03	18	24
Hail	06	02	08	06	03	09	17
Northern	03	01	04	06	00	06	10
Jazan	38	53	91	87	18	105	196
Najran	08	05	13	17	07	24	37
Al-Bahah	09	00	09	10	03	13	22
Al-Jouf	02	01	03	03	00	03	06
Qurayyat	04	01	05	10	00	10	15
Qunfudah	05	01	06	10	01	11	17
Total	545	291	836	1088	364	1452	2543

* Number of cases per 100,000.

**Fig.1:**
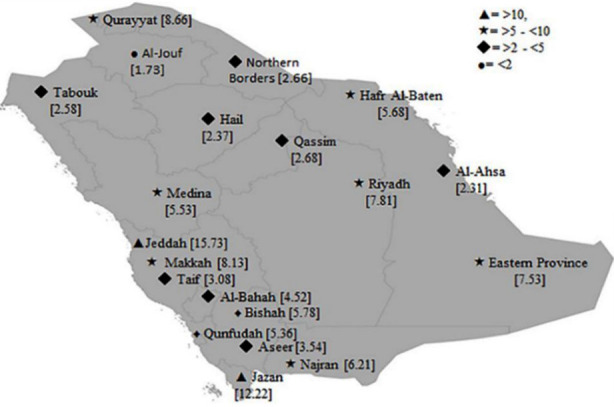
The Incidence Rate/100,000 of pulmonary tuberculosis in different regions of Kingdom of Saudi Arabia. Regions were divided into 4 different categories and indicated by symbols: ▲= incidence rate >10/100,000, *= >5 - <10/100,000, ♦ = >2 - <5/100,000 and •= <2/100,000.

Regarding the incidence of infection in non-Saudi nationals, the highest incidence rate was recoded in Jeddah, followed by Riyadh. Also, the incidence rate was significantly higher (p ≤0.001) in males than in females representing 73.6% and 71.3%, respectively. The incidence of infections in males amongst the non-Saudi nationals was higher than that in females in all cities without exceptions. The map in Fig.1 depicts the incidence of pulmonary TB in different cities; the symbols code indicates the magnitude of incidences and classifies them into four different rates.

### Assessment of Risk Factors:

**T**he distribution of infections in different age groups in males and females, Saudi and non-Saudi nationals. Table-II Amongst male Saudis, the highest percentages of infections were reported in the age range <15, 15-25 and 25-35 in order. In Saudi females, the highest percentage of infections were in age groups 45-55, followed by 35-45. In this respect, there was a statistically significant difference between the number of infections in males and females in the above-mentioned age groups (*p* *= 0.001*).

**Table-II:** Age-wise incidence of Pulmonary Tuberculosis among Saudi and non-Saudi population.

Age Group	Age scale	Saudi n (%)		p-values	Non-Saudi n (%)		p-values
	
		Male	Female		Male	Female	
Group I	< 15	54(100)	0(0)	0.001	0(0)	0(0)	0.001
Group II	15 - < 25	424(95)	21(5)		5(100)	0(0)	
Group III	25 - < 35	318(56)	250(44)		162(100)	0(0)	
Group IV	35 - < 45	4(19)	17(81)		465(100)	0(0)	
Group V	45 - < 55	0(0)	3(100)		329(99)	4(1)	
Group VI	55 - < 65	0(0)	0(0)		112(46)	131(54)	
Group VII	≥ 65	0(0)	0(0)		15(6)	229(94)	

The highest number of infections amongst male non-Saudi nationals was recorded in the age groups 25-35 and 35-45, followed by 45-55. On the other hand, the highest female infections were in ≥ 65 then 55-65 age groups. Likewise, the difference was statistically significant between the number of infections in male and female non-Saudi nationals in these age groups (*p=0.001*).

## DISCUSSION

In the current study, the overall rate of incidence of pulmonary TB found to be 7.61/ 100,000 in Saudi Arabia for the year 2018. Globally, the TB incidence rate is falling by roughly 2% a year.^7^,^18^ In 2019, the incidence of TB in Saudi Arabia was 9.9 cases/100,000. The incidence of tuberculosis in Saudi Arabia fell gradually from 19 cases/100,000 in 2000, to 9.9 cases/100,000 in 2019.^6^ The difference in the incidence of TB between genders could be attributed to sex hormones that may be responsible for modulating the immune response, which is necessary for fighting the pathogen and other infectious diseases.^19^,^20^ Recently, the mortality rate of TB was reported to be 22/1000 person-years being higher in males and elderly. Also, it has been suggested that the reactivation of latent MTB infection in elderly age could be due to immunosenescence and co-morbidities.^21^,^22^

In the current study, the number of cases of TB in males amongst the non-Saudi nationals was found to be higher than that in Saudi nationals; most likely because most of them came from countries with a high burden of TB.^6^ The highest number of infections amongst male non-Saudi nationals was recorded in the age groups 25-35 and 35-45, followed by 45-55; this might be explained by the socioeconomic status, where in these age-groups there are a lot of unskilled workers who tend to live in crowded shelters, with poor sanitation and hygiene.^23^ These conditions make them susceptible for reactivation of latent TB. Expatriates arriving to the kingdom for work are not screened for TB, except for house workers and health professionals.^24^ The highest incidence rate of TB amongst non-Saudis, which was recoded in Jeddah, followed by Riyadh, may be associated with the higher proportion of non-Saudis in urban areas and industrial cities. Jeddah receives more than ten million foreign visitors every year for Hajj and Umrah.^25^

Another Saudi study reported low prevalence of TB and multidrug resistant TB over 17-year period in a single center.^26^ Having indicated that Saudi Arabia is a global destination and with continuous influx and outflux of people in and out from the country, there might be a chance of Saudi Arabia to become a spread point of infection in case TB incidence rises in a steady state. Therefore, we have to notify the readers that gulf health council (GHC) members that include Saudi Arabia have the highest prevalence of diabetes in the world, where the International Diabetic Federation (IDF) ranked five members of the GHC amongst the top 10 countries with high prevalence of diabetes. In this context, Saudi Arabia ranked third in the world by the IDF. Since diabetes has been found to increase TB risk, the high prevalence of diabetes in Saudi Arabia should be a warning sign.^9^

## CONCLUSION

There was a significant difference between males and females regarding contracting the TB infection amongst both the Saudi and the non-Saudi nationals suggesting an association between gender and the increased risk of infection. Likewise, analysis of infection in different age groups suggested that the incidence rates in Saudis were recorded in younger age groups than in the non-Saudis, which is surprising. Importantly, we do not know whether non-Saudis are residents or visitors as it was not mentioned in the MOH database. In this respect, there should be a proper screening program for high-risk groups as well as a plan to investigate the risk factors that might be associated with the increased incidence of pulmonary TB. Altogether, these analyses and approaches would help health authority decision makers to establish policies and procedures to limit the infection and apply appropriate precautionary measures. To this end, this study analyzed the data of pulmonary TB incidence rates in 2018 where the data for other years were not reported on the MOH data repository. It would be more informative if we could obtain data for a longer period for the study to be more conclusive.
